# Effects of Slow Oscillatory Transcranial Alternating Current Stimulation on Motor Cortical Excitability Assessed by Transcranial Magnetic Stimulation

**DOI:** 10.3389/fnhum.2021.726604

**Published:** 2021-09-13

**Authors:** Asher Geffen, Nicholas Bland, Martin V. Sale

**Affiliations:** ^1^School of Health and Rehabilitation Sciences, The University of Queensland, St Lucia, QLD, Australia; ^2^Queensland Brain Institute, The University of Queensland, St Lucia, QLD, Australia; ^3^School of Human Movement and Nutrition Sciences, The University of Queensland, St Lucia, QLD, Australia

**Keywords:** transcranial alternating current stimulation, transcranial magnetic stimulation, entrainment, plasticity, neural oscillations

## Abstract

Converging evidence suggests that transcranial alternating current stimulation (tACS) may entrain endogenous neural oscillations to match the frequency and phase of the exogenously applied current and this entrainment may outlast the stimulation (although only for a few oscillatory cycles following the cessation of stimulation). However, observing entrainment in the electroencephalograph (EEG) during stimulation is extremely difficult due to the presence of complex tACS artifacts. The present study assessed entrainment to slow oscillatory (SO) tACS by measuring motor cortical excitability across different oscillatory phases during (i.e., online) and outlasting (i.e., offline) stimulation. 30 healthy participants received 60 trials of intermittent SO tACS (0.75 Hz; 16 s on/off interleaved) at an intensity of 2 mA peak-to-peak. Motor cortical excitability was assessed using transcranial magnetic stimulation (TMS) of the hand region of the primary motor cortex (M1_HAND_) to induce motor evoked potentials (MEPs) in the contralateral thumb. MEPs were acquired at four time-points within each trial – early online, late online, early offline, and late offline – as well as at the start and end of the overall stimulation period (to probe longer-lasting aftereffects of tACS). A significant increase in MEP amplitude was observed from pre- to post-tACS (paired-sample *t*-test; *t_29_* = 2.64, *P* = 0.013, *d* = 0.48) and from the first to the last tACS block (*t_29_* = −2.93, *P* = 0.02, *d* = 0.54). However, no phase-dependent modulation of excitability was observed. Therefore, although SO tACS had a facilitatory effect on motor cortical excitability that outlasted stimulation, there was no evidence supporting entrainment of endogenous oscillations as the underlying mechanism.

## Graphical Abstract

Thirty healthy participants received 60 trials of intermittent SO (0.75 Hz) tACS (1 trial = 16 s on + 16 s off) at an intensity of 2 mA. Motor cortical excitability was assessed using TMS-induced MEPs (blue waveforms) acquired across different oscillatory phases during (i.e., online; red arrows) and outlasting (i.e., offline; green arrows) tACS, as well as at the start and end of the stimulation session (blue arrows). Mean MEP amplitude increased by ∼41% from pre- to post-tACS (*P* = 0.013); however, MEP amplitudes were not modulated with respect to the tACS phase.

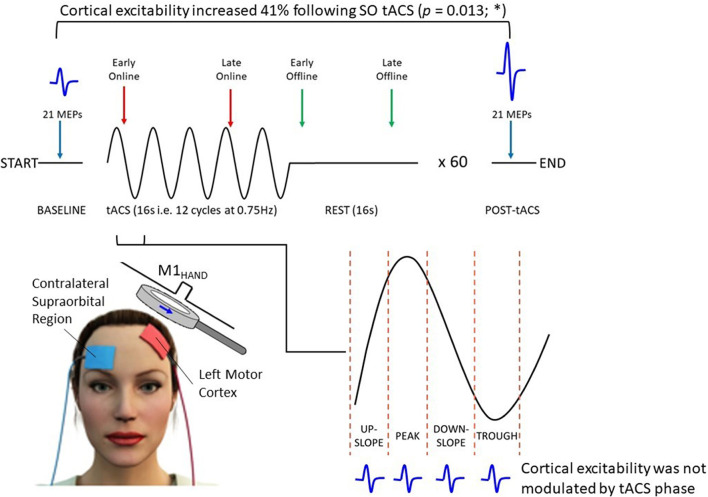

## Introduction

Neural oscillations (i.e., cyclic fluctuations in neuronal excitability) are proposed to provide phase-dependent temporal regulation of neural information processing (Buzsáki, 2006). In order to explore the functional relationships between neural oscillations and behavior in normal brain function, rhythmic subtypes of transcranial electrical stimulation (tES) have been used to attempt to modulate endogenous neural oscillatory activity experimentally. tES has already been shown to influence various aspects of behavior and cognition by modulating the power of specific neural oscillations known to be associated with such tasks (for reviews see, Antal and Paulus, 2013; Herrmann et al., 2016; Vosskuhl et al., 2018; Bland and Sale, 2019). However, despite promising findings of behavioral effects induced by transcranial alternating current stimulation (tACS), the neurophysiological mechanisms behind these effects are still not well understood.

Converging evidence from animal (e.g., Krause et al., 2019; see also review by Reato et al., 2013), computational (Reato et al., 2010; Ali et al., 2013; Huang et al., 2021), and human studies (Helfrich et al., 2014a,b; Witkowski et al., 2016) suggests that during stimulation, tES may be able to entrain (i.e., synchronize) endogenous neural oscillations with respect to the frequency and phase of the exogenously applied current. However, unlike the well-documented immediate (online) effects of tES, there is less agreement regarding the magnitude and duration of post-stimulus (offline) effects (for review see, Veniero et al., 2015). These offline effects cannot be fully explained by a direct continuation of online entrainment (referred to as entrainment “echoes”), since these “echoes” only persist for a few oscillatory cycles following cessation of stimulation (Marshall et al., 2006; Thut et al., 2011; Hanslmayr et al., 2014; van Bree et al., 2021). Therefore, longer-lasting offline effects (referred to as aftereffects) lasting up to 70 min are likely to reflect mechanisms other than entrainment *per se* (e.g., synaptic plasticity) (Neuling et al., 2013; Veniero et al., 2015; Vossen et al., 2015; Kasten et al., 2016).

The effects of tES on endogenous oscillatory activity have traditionally been quantified in humans using electroencephalography (EEG; Marshall et al., 2006; Kirov et al., 2009; Jones et al., 2018; Ketz et al., 2018). However, observing entrainment in the EEG concurrently with tES is extremely difficult due to the presence of complex artifacts (Noury et al., 2016; Noury and Siegel, 2017; Kasten and Herrmann, 2019) and we have therefore used an alternative method to assess entrainment of endogenous neural oscillations by tES. Single-pulse transcranial magnetic stimulation (TMS) is a form of non-invasive brain stimulation that can be used to indirectly probe the excitability of neocortical networks with high spatiotemporal precision of the order of millimeters and milliseconds (Hallett, 2007). When applied to the hand area of the primary motor cortex (M1_HAND_), each TMS pulse induces a motor evoked potential (MEP) in the contralateral target muscle, the amplitude of which can then be measured using electromyography (EMG; Barker et al., 1985). These MEP amplitudes provide an indirect measure of motor cortical excitability with good topographical specificity (Di Lazzaro et al., 2004; Hallett, 2007; Ilmoniemi and Kicić, 2010; Bergmann et al., 2012). By applying TMS pulses within a particular oscillatory phase of tES (referred to as phase-dependent stimulation), TMS can be used to assess entrainment of endogenous neural oscillations by tES (i.e., whether motor cortical excitability is modulated with respect to the phase of tES; Raco et al., 2016; Zrenner et al., 2018; Schaworonkow et al., 2019). Importantly, the artifact issues of EEG are not present with TMS–EMG measures, thus allowing for an unambiguous investigation of the phasic effects of tES on motor cortical excitability.

Because we wanted to probe the phase-specific effects of tES, we chose to apply tES at a low frequency to allow MEP sampling across the different phases of stimulation. In this manner, the phase-cycle of low-frequency tACS could be conceptualized as representing alternating periods of classic “anodal” and “cathodal” transcranial direct current stimulation (tDCS), on which much earlier work has focused (Antal and Paulus, 2013; Reato et al., 2013; Liu et al., 2018; Bland and Sale, 2019). Therefore, in the present investigation, we chose to examine the online and offline effects of slow oscillatory (SO; 0.75 Hz) tACS on motor cortical excitability using TMS.

Slow oscillations are typically prevalent during slow-wave sleep and play an important role in sleep-dependent consolidation of motor learning (Marshall and Binder, 2013). Despite the lack of endogenous SO activity during wakefulness, anodal SO tDCS during wakefulness has been shown to increase endogenous SO EEG power with relatively short-lasting offline effects (<1 min) (Kirov et al., 2009), though the exact duration of these offline effects was not thoroughly assessed. However, it is important to note that these increases in SO power were more restricted topographically to the prefrontal cortex (the predominant source of endogenous slow oscillations during sleep) and were less pronounced than those observed following SO tDCS applied *during* sleep (Marshall et al., 2006). Furthermore, due to the previously mentioned complexity of tES artifacts in the EEG, the authors could not determine whether these localized increases in EEG SO power were in fact due to the entrainment of slow oscillations by SO tDCS. Therefore, it remains unclear whether slow oscillations can be reliably entrained in the awake brain at intensities typical of tES (i.e., 1–2 mA).

Anodal SO tDCS during wakefulness has also been shown to induce lasting increases in motor cortical excitability that persist beyond stimulation (Bergmann et al., 2009; Groppa et al., 2010). However, due to the anodal component (i.e., positive current offset) of this stimulation—which in itself can cause an increase in cortical excitability (Nitsche and Paulus, 2000; Nitsche et al., 2007; Bergmann et al., 2009)—it cannot be concluded that these effects are a direct result of the influence of the applied slow oscillations. tACS has a significant technical advantage over tDCS in this regard, since it has no DC offset (i.e., an average current of 0 mA). Despite this, the effects of SO tACS on motor cortical excitability have not been thoroughly examined in previous literature. Antal et al. (2008) found no significant changes in motor cortical excitability following SO (1 Hz) tACS; however, their stimulation protocol was suboptimal for inducing changes in endogenous oscillatory activity due to the low stimulation intensity (0.4 mA; Reato et al., 2010; Huang et al., 2017) and constant rather than intermittent application of tACS (Jones et al., 2018; Ketz et al., 2018).

The aims of this study were: (1) to investigate the online effects of SO tACS applied intermittently at high intensity on motor cortical excitability; (2) to determine if tACS-induced changes in motor cortical excitability persist beyond each trial of stimulation (i.e., entrainment echoes) as well as beyond the total stimulation period (i.e., offline aftereffects).

It was hypothesized that SO tACS will induce SO-like sinusoidal changes in motor cortical excitability that correspond with the tACS phase, with high MEP amplitudes at oscillatory peaks and low amplitudes at oscillatory troughs, supporting online entrainment. Secondly, that sinusoidal changes in motor cortical excitability will persist for a few oscillatory cycles immediately following each trial of stimulation, thus demonstrating entrainment echoes. Thirdly, that motor cortical excitability will increase over the total duration of stimulation (although this relationship may not necessarily be linear), and this increase will be sustained beyond the total stimulation period (i.e., offline aftereffects).

## Materials and Methods

### Subjects

Forty-one neurologically healthy, right-handed participants (17 male, aged 24 ± 4 years) were recruited by advertisement, although 11 participants were excluded from the final analysis (see “MEP screening” below) leaving a sample size of 30 participants. All participants completed a safety screening questionnaire (Keel et al., 2001) and provided a written statement of informed consent prior to commencing the experiment. The exclusion criteria for participants included: personal or family history of epilepsy/seizures, medication that could affect seizure threshold, history of brain injury/condition (e.g., stroke, concussion, etc.), implanted devices or metal in the head, frequent or severe headaches, or current pregnancy. Approval was granted by The University of Queensland Human Research Ethics Committee.

### Quantification of Motor Cortical Excitability Using TMS

Motor cortical excitability was assessed by measuring TMS-induced MEP amplitudes that were recorded from the target muscle using surface EMG. The target site for the TMS was the left M1_HAND_ region, specifically the region associated with the *abductor pollicis brevis* (APB), a large thumb muscle.

### Experimental Setup

#### EMG

Participants were seated comfortably in a chair and their right forearm placed on a foam mat with their forearm supinated. EMG activity of the APB muscle was recorded using disposable surface electrodes (H124SG 30 mm × 24 mm). The active electrode was placed over the APB muscle belly, the reference electrode was placed over the first metacarpophalangeal joint, and the ground electrode was placed on the anterior surface of the wrist.

#### TMS

Transcranial magnetic stimulation pulses were applied to the left M1_HAND_ region using a Magstim Double 70 mm Remote Control Coil charged by a Magstim 200^2^ stimulator (Magstim, United Kingdom). The individual location of the left M1_HAND_ region as well as the TMS intensity were determined for each participant using manual TMS “hot-spotting” (Rossini et al., 1994). This involves systematically adjusting the position of the TMS coil on the participant’s head whilst also adjusting the stimulation intensity until MEPs are consistently induced (i.e., in at least five out of ten successive trials) with amplitudes within a desired range (in our case, 0.5–1.5 mV; see Cuypers et al., 2014; Thies et al., 2018; Ogata et al., 2019). The location of the left M1_HAND_ region was then marked on the participant’s scalp using an erasable marker.

#### tACS

Transcranial alternating current stimulation was applied using a NeuroConn DC Stimulator Plus. The 42 × 45 mm tACS pad electrodes were applied to the scalp using a classical M1-contralateral supraorbital region electrode montage (see Heise et al., 2016), with the target electrode placed over the left M1 (which roughly corresponds with the EEG coordinate C3) and the return electrode placed over the contralateral supraorbital region. However, the electrode targeting the left M1 was not placed directly over the TMS hotspot itself, but rather ∼2 cm posterolateral to the hotspot (which roughly corresponds with Cp3). This slight increase in inter-electrode distance is thought to reduce current shunting through the scalp and cerebrospinal fluid, thus, maximizing current density at the target site and increasing the effectiveness of the tACS (Faria et al., 2011). Before attaching the electrodes, the scalp was rubbed with ethanol (70%) and Ten20 conductive paste was applied to the electrodes to minimize resistance between the electrodes and scalp.

#### Recording tACS Output

The tACS output was recorded using disposable surface electrodes (H124SG 30 mm × 24 mm). These electrodes were placed over the tACS pad targeting the supraorbital region and on the left side of the forehead, and referenced to the nose tip. The tACS artifact was used to synchronize stimulation with the computer used for MEP acquisition (i.e., such that each probe by TMS was timed with respect to the phase of tACS).

#### Data Collection

All surface electrode measurements (i.e., APB EMG and tACS output electrodes) were acquired (1 KHz sampling rate; 20–1000 Hz band pass filtering) via an electrode adaptor (Model CED1902-1 1/2), before being amplified by a CED1902, and finally recorded by a CED1401 MICRO3 (Cambridge Electronic Designs, Cambridge, United Kingdom). TMS triggers were directly recorded by the CED1401 MICRO3. All data were then transferred from the CED1401 MICRO3 to a PC and saved via Signal (Ver. 6.04) software (Cambridge Electronic Designs, Cambridge, United Kingdom), before being exported to MATLAB (Ver. R2019a) and subsequently JASP (Ver. 0.14.1.0) for analysis.

### Experimental Procedure

#### tACS Paradigm

**FIGURE 1:**
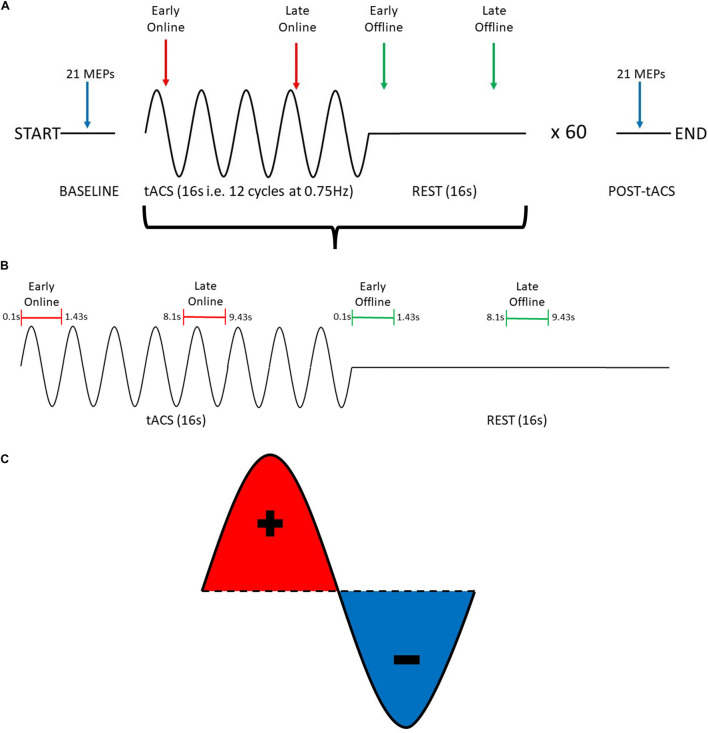
Summary of experimental procedure for probing changes in motor cortical excitability induced by SO tACS. **(A)** The experimental session consisted of a 16-s tACS period (represented as sine waves) followed by a 16-s rest period (represented as flat lines), repeated 60 times for a total of ∼32 min. To probe how tACS-induced changes in motor cortical excitability evolve over time, 21 TMS-induced MEPs were acquired at the start and the end of the experimental session (blue arrows), 2 MEPs were acquired at each tACS period (red arrows), and 2 MEPs were acquired at each rest period (green arrows). **(B)** Within each tACS trial (i.e., tACS + rest period), MEPs were acquired at 4 distinct time-points (1 MEP per time-point): early online, late online, early offline, and late offline. **(C)** tACS alternates polarity between the anode and cathode to produce a sinusoidal current with both positive (red) and negative (blue) peaks.

Participants received 60 trials of tACS, with each trial consisting of 16 s (12 cycles at 0.75 Hz) of tACS at an intensity of 2 mA (“Online”), followed by 16 s of no tACS (“Offline”), for a total of 16 min of tACS and 16 min of no tACS (Figure 1A). The entire stimulation period was divided into 3 blocks (∼10 min comprising 20 trials each), with 5-min rest periods (no tACS or TMS delivered) between blocks.

#### TMS Paradigm

To examine the online and offline effects of SO tACS on motor cortical excitability, MEPs were acquired at 4 time-points within each trial (1 MEP per time-point): early online (0.1–1.43 s after tACS starts), late online (8.1–9.43 s after tACS starts), early offline (0.1–1.43 s after tACS ends), and late offline (8.1–9.43 s after tACS ends) (Figure 1B). Therefore, 60 MEPs were acquired for each time-point (i.e., once each per trial).

To examine if the effects of SO tACS on motor cortical excitability are specific to the tACS phase (both online and offline), sufficient MEPs need to be acquired across the different phases of the tACS (Zoefel et al., 2019). This was achieved by implementing a “jitter” (i.e., a randomized time delay that covers the length of a single tACS cycle) to the delivery of TMS so that the delivery was not locked to a specific phase of the tACS (i.e., the timing of TMS delivery was random across the tACS cycle), and thus, TMS pulses were approximately uniformly delivered across the different phases across the entire stimulation block (Figure 1C).

To examine the cumulative effects of the entire tACS paradigm on motor cortical excitability, 21 TMS-induced MEPs were acquired both at baseline and at the end of the entire period of tACS delivery (Figure 1A). TMS was delivered at ∼0.2 Hz.

### Statistical Analysis

#### Data Transformation

The first MEP for each data set (as well as the first MEP after each of the rest periods) was always excluded, since initial MEP amplitudes may be larger (Brasil-Neto et al., 1994) and more variable (Schmidt et al., 2009) than subsequent MEPs, which can impact the reliability of TMS measures of cortical excitability. Further, individual MEPs were excluded if voluntary pre-MEP EMG activity was detected in the 500 ms prior to TMS delivery (2.72% of MEPs excluded). Finally, participants with mean pre-tACS amplitudes less than 0.5 or greater than 1.5 mV were excluded from the final analysis (11 participants excluded). This is because excessively small or large pre-tACS MEPs may have introduced floor and ceiling effects, respectively (Cuypers et al., 2014).

**FIGURE 2:**
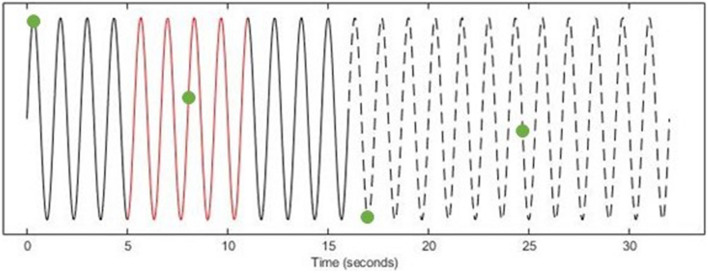
Determining tACS phase at TMS triggers (EXAMPLE ONLY). Using the tACS-output recording (Solid Line), a 6-s window (Red) of the instantaneous phase (*centered* on each late online trigger) was computed. The computed phase was then extrapolated both forward into the offline period (Dashed Line) and backward to the early online triggers (Solid Line) and the phase was computed at each of the other time-points (Green Dots).

The TMS triggers were then automatically categorized into their respective time-points (i.e., early online, late online, early offline, and late offline–see Figure 1B) and the “late online” triggers were used to calculate the tACS phase, since these triggers are the only ones where tACS was present both before and after TMS was applied, thus, providing the most reliable estimate of tACS phase. If tACS-induced phase entrainment persists beyond stimulation, we would expect the tACS phase to continue into the offline period. To assess this, the computed phase for the late online triggers was extrapolated (both forward and backward) and its values computed at each of the other time-points (Figure 2).

#### Data Analysis

To determine if the effects of SO tACS on motor cortical excitability are specific to the tACS phase a permutation analysis was performed (Zoefel et al., 2019). This analysis was performed separately for each of the four time-points (∼60 MEPs per time-point per participant) as well as for all online and offline MEPs (∼120 MEPs online/offline per participant).

For the permutation analysis, an ideal (i.e., best-fitting, 0.75 Hz) sinusoidal model was fitted to each participant’s observed MEP amplitudes based on their phase for each of the four time-points (Bland and Sale, 2019), and the amplitudes of these models were summed. Because these models were fitted with bias (baseline), amplitude, and phase all free to vary across participants, there was no need for alignment of individual “preferred” phase. The MEP amplitudes were then shuffled with respect to their phases to form a surrogate distribution of expected amplitudes under the null hypothesis (which assumes that the MEP amplitudes are not modulated with respect to phase). Next, ideal sinusoidal models were fitted to the shuffled data, and the amplitudes of these shuffled models were summed. This process was repeated for a total of 1000 permutations per participant. The true and shuffled summed amplitudes were then compared. In this analysis, the *P*-value is the proportion of shuffled summed amplitudes exceeding the true sum of amplitudes, remembering that under the null hypothesis the amplitude of these sinusoidal models should be small (i.e., closer to zero). Because the permutation procedure disrupts any phasic effects that may be present, the shuffled MEPs act as a negative control for the true MEPs, and thus, the permutation analysis does not require a sham stimulation condition as a negative control.

To determine if there was a significant difference in mean MEP amplitudes between the pre- and post-tACS measurements, a paired sample *t*-test was performed. To determine if there was a significant difference in mean MEP amplitudes between the online and offline measurements or between the three tACS blocks, a two-way repeated measures ANOVA was performed with *stimulation* (online, offline) and *block* (1, 2, 3) as the two repeated measures factors. *Post hoc t*-tests (corrected for multiple comparisons using Holm’s method) were then performed to compare the individual groups against each other. To examine how offline changes in MEP amplitudes evolve throughout the tACS period, mean MEP amplitudes for the pre- and post-tACS MEPs and the offline MEPs of each tACS block were compared using a one-way repeated measures ANOVA with *time* as the repeated measures factor (5 levels). Again, *post hoc t*-tests were then performed to compare the individual groups against each other. For the repeated measures ANOVAs, standardized effect sizes for any significant differences were calculated as η^2^ values. For the *post hoc t*-tests, standardized effect sizes for any significant differences were calculated as Cohen’s *d* values.

## Results

### Phase-Specificity of tACS Effects

**FIGURE 3:**
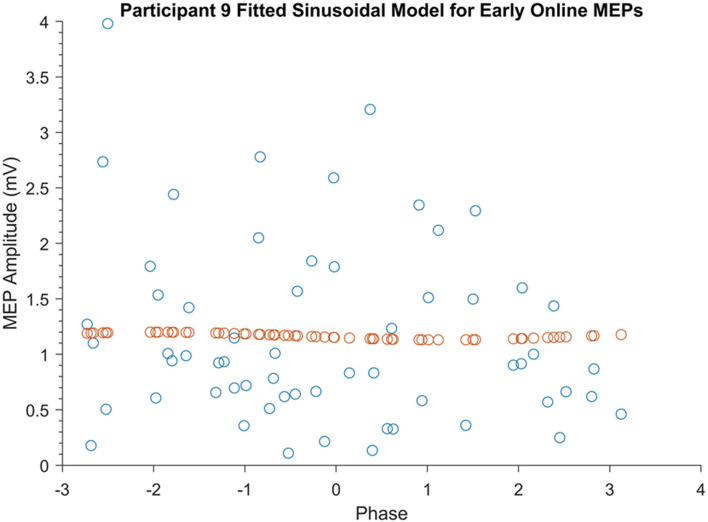
Example of a participant’s fitted sinusoidal model for early online MEPs. Blue dots represent Participant 9’s early online MEPs sorted according to tACS phase. Orange dots represent the fitted sinusoidal model for these MEPs.

The phase-specificity of acute changes in motor cortical excitability induced by SO tACS was assessed by a permutation analysis. Ideal sinusoidal models were fitted to each participant’s observed MEP amplitudes based on their phase for each TMS time-point (∼60 MEPs per time-point per participant, see Supplementary Figure S1) and the amplitudes of these models (see Supplementary Figure S2) were summed. An example of one of these fitted sinusoidal models is shown in Figure 3. The true sum of amplitudes was then compared against the summed amplitudes of 1000 permutations of the MEP amplitudes, with *P*-values representing the proportion of shuffled summed amplitudes exceeding the true sum of amplitudes. The permutation analysis did not reveal significant phase-specific modulation of motor cortical excitability at any of the four TMS time-points (*P* = 0.86, 0.81, 0.21, and 0.70 for early online, late online, early offline, and late offline, respectively). Combining the early and late online/offline MEPs (∼120 MEPs online/offline per participant) also failed to reveal any significant phase-specific modulation of motor cortical excitability online or offline to tACS (*P* = 0.89 and 0.90, respectively).

### Cumulative Effects of SO tACS on Motor Cortical Excitability

**FIGURE 4:**
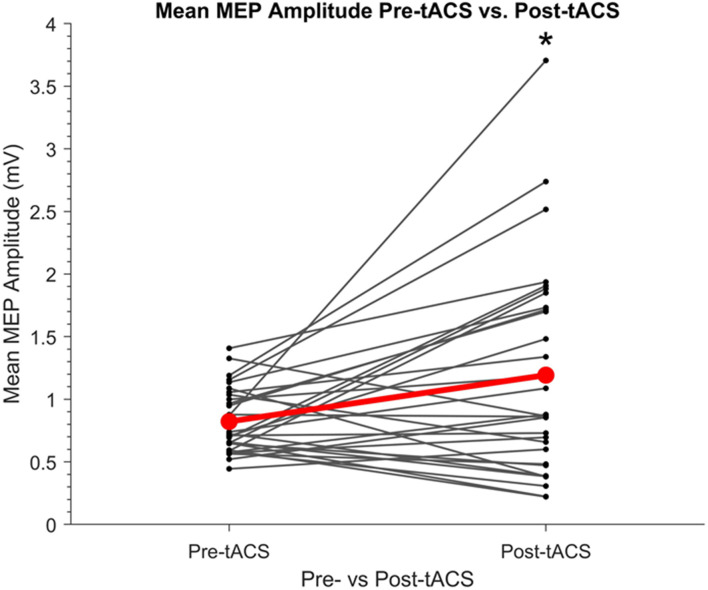
Individual mean amplitudes of TMS-induced MEPs before and after SO tACS. Points represent each participant’s mean MEP amplitude from 20 TMS-induced MEPs (per participant) acquired before (pre-tACS) and after (post-tACS) receiving 60 “trials” (∼32 min) of SO tACS, with each trial consisting of 16 s of tACS (12 cycles at 0.75 Hz) followed by 16 s of rest. Individual differences in mean MEP amplitude are represented by the solid lines. The global mean MEP amplitude for each group is represented by a red point, with the global mean difference from pre- to post-tACS represented by a red line connecting the two red points. A significant increase in MEP amplitude from pre-tACS to post-tACS was reported (paired sample *t*-test *t*_29_ = 2.64, *P* = 0.013, *d* = 0.48; indicated by *); *n* = 30.

The cumulative effects of the tACS paradigm on motor cortical excitability was assessed by comparing mean MEP amplitudes pre- and post-tACS. As shown in Figure 4, MEP amplitudes were found to be significantly greater post-tACS (mean = 1.19 mV ± 0.84) compared to pre-tACS (mean = 0.82 mV ± 0.26) (*t_29_* = 2.64, *P* = 0.013, *d* = 0.48).

**FIGURE 5:**
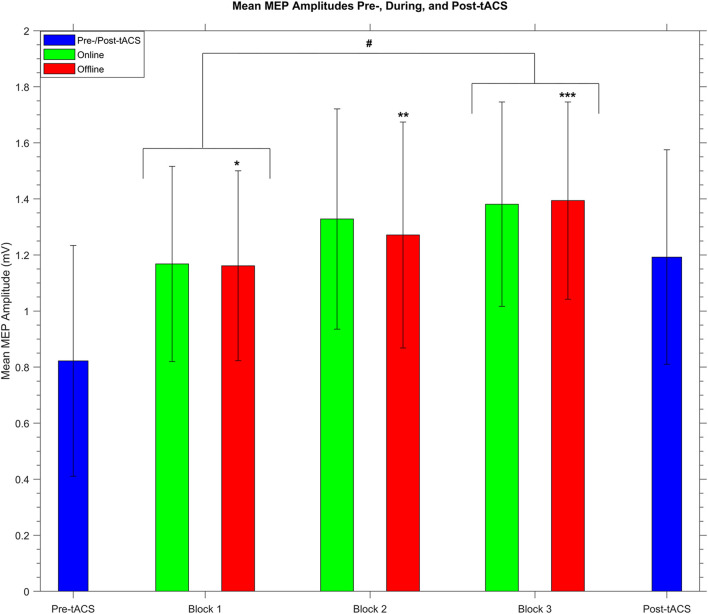
Group mean MEP amplitudes before, during, and after receiving SO tACS. Blue bars represent mean MEP amplitudes (with within-subjects error bars) from 20 TMS-induced MEPs (per participant) acquired before (pre-tACS) and after (post-tACS) receiving 60 “trials” (∼32 min, split into 3 20-trial “blocks”) of SO tACS, with each trial consisting of 16 s of tACS (online, 12 cycles at 0.75 Hz) followed by 16 s of rest (offline). Mean MEP amplitudes from 40 MEPs acquired during the online and offline periods of each block are represented by green and red bars, respectively. There was a significant increase in mean amplitude from the 1st to the 3rd block (t_29_ = −2.93, *P* = 0.02, *d* = 0.54; indicated by #). Furthermore, offline mean amplitudes were significantly greater than pre-tACS mean amplitudes for all three blocks (t_29_ = −3.24, −4.02, and −4.56; *P* = 0.024, 0.003, and <0.001; *d* = 0.59, 0.73, and 0.83 for blocks 1, 2, and 3, respectively; indicated by *, **, and ***, respectively); *n* = 30.

Because there was a significant increase in mean MEP amplitude from pre- to post-tACS, the question arose of whether this overall change in MEP amplitude occurred gradually over time within the stimulation period. We therefore compared online and offline mean MEP amplitudes across each of the three tACS blocks. A two-way repeated measures ANOVA revealed a significant main effect of *block* (*F*_2_,_58_ = 3.77, *P* = 0.03, η^2^ = 0.1) but no main effect of *stimulation* (*F*_1_,_29_ = 0.65, *P* = 0.43) and no significant *block* × *stimulation* interaction (*F*_2_,_58_ = 1.29, *P* = 0.28). As shown in Figure 5, subsequent *post hoc t*-tests confirmed a significant increase in MEP amplitudes between the 1st (mean = 1.19 mV ± 0.72) and 3rd (mean = 1.46 mV ± 0.94) blocks (*t*_29_ = −2.93, *P* = 0.02, *d* = 0.54), whereas there were no significant differences between the 1st and 2nd (mean = 1.33 mV ± 0.79) blocks or between the 2nd and 3rd blocks (*t*_29_ = −1.6 and −1.04, respectively, *P* = 0.24 and 0.31, respectively). This suggests a gradual build-up of cortical excitability from tACS.

We also wished to compare mean MEP amplitudes from the offline periods of each block against each other as well as against the pre- and post-tACS mean amplitudes. A one-way repeated measures ANOVA revealed a significant main effect of *time* (*F*_4_,_116_ = 7.84, *P* < 0.001, η^2^ = 0.21). As shown in Figure 5, subsequent *post hoc t*-tests revealed that offline mean MEP amplitudes for all 3 blocks were significantly greater than pre-tACS mean amplitudes (*t*_29_ = −3.24, −4.02, and −4.56; *P* = 0.024, 0.003, and <0.001; *d* = 0.59, 0.73, and 0.83 for blocks 1, 2, and 3, respectively). However, no other significant differences in offline mean amplitude were reported when comparing the blocks against each other or against the post-tACS amplitudes, although the difference between the 1st and 3rd blocks was only marginally insignificant (*t*_29_ = −2.81, *P* = 0.06).

It is worth mentioning that widening the exclusion criteria for participants based on their pre-tACS mean MEP amplitudes (0.5–1.5 mV to 0.4–2 mV) did not affect the significance of the cumulative or phase-specific effects of SO tACS in the present study, despite increasing the sample size from 30 to 36 participants.

## Discussion

Although there has been a plethora of studies in the last decade reporting behavioral, perceptual, and electrophysiological effects induced by tACS (for reviews see, Antal and Paulus, 2013; Herrmann et al., 2016; Vosskuhl et al., 2018; Bland and Sale, 2019), the mechanisms underlying these effects remain only rudimentarily understood. In the present study, we aimed to probe SO tACS-induced entrainment of endogenous slow oscillations both online and offline by assessing motor cortical excitability across different oscillatory phases using TMS-induced MEP amplitudes. We also assessed the cumulative effects of SO tACS on motor cortical excitability by comparing mean excitability pre- and post-stimulation as well as comparing mean excitability across stimulation blocks.

Regarding the cumulative effects of SO tACS, we present the first evidence of enhanced motor cortical excitability induced by SO tACS in the awake brain, with a significant increase in TMS-induced MEP amplitudes from pre- to post-tACS as well as from the 1st to the 3rd tACS block. Excitability increases induced by anodal SO tDCS have been reported previously (Bergmann et al., 2009; Groppa et al., 2010). However, compared to the SO tDCS study by Bergmann et al. (2009), the present study using SO tACS demonstrated a greater MEP amplitude increase (45.05% vs. 22%) despite shorter stimulation periods (16 s vs. 30 s), shorter total duration of stimulation (16 min vs. 17.5 min), longer rest periods between trials (16 s vs. 5 s) and two additional 5-min rest periods. Critically, due to the lack of an anodal component for tACS, this excitatory effect cannot be attributed to a general depolarization of cortical motor neurons and is thus driven by some other factor.

It is theoretically possible that the cumulative increase in motor cortical excitability was associated with an entrainment of endogenous slow oscillations (Marshall et al., 2006; Kirov et al., 2009; Jones et al., 2018; Ketz et al., 2018). However, the acute effects of stimulation did not appear to be dependent on the tACS phase, with the permutation analysis providing no evidence for phase-specific modulation of motor cortical excitability at any of the four TMS time-points (i.e., early/late online/offline) or for the combined online/offline MEPs.

The most likely explanation for the lack of an entrainment effect in the present results is that endogenous slow oscillations are not prevalent enough in the wake brain to be effectively entrained by SO tACS. This conclusion is in line with previous SO tDCS/tACS studies (Marshall et al., 2006; Bergmann et al., 2009; Kirov et al., 2009; Groppa et al., 2010; Jones et al., 2018; Ketz et al., 2018) as well as tACS studies using different stimulation frequencies (Antal et al., 2008; Kanai et al., 2008; Ali et al., 2013) that found stimulation to be most effective when the frequency of the exogenously applied oscillations closely matches the frequency of the predominant endogenous oscillations. These findings suggest that network resonance is a key underlying mechanism by which tACS modulates large-scale cortical network activity. These resonance dynamics are characterized by a phenomenon called an “Arnold Tongue,” where the current intensity required to induce a particular oscillation increases the more the frequency of that oscillation deviates from the resonant (eigen) frequency of the network (Ali et al., 2013; Thut et al., 2017; Liu et al., 2018).

If slow oscillations were in fact entrained by SO tACS, these entrained slow oscillations would likely be of a smaller magnitude than those that naturally occur during sleep (Marshall et al., 2006; Kirov et al., 2009; Jones et al., 2018; Ketz et al., 2018). Therefore, it is possible that the sensitivity of the current permutation analysis was insufficient to detect such a subtle entrainment effect. The sensitivity of our permutation analysis may have been impacted by the relatively low number of MEPs (60 MEPs per time-point per participant), which we know from simulation studies impacts detectability (Zoefel et al., 2019). However, it is important to note that the number of MEPs we could acquire was limited by a number of practical considerations, including coil recharge time, coil heating, and session length, whereas simulation MEP numbers are unencumbered by practical limitations. Because the number of MEPs we can acquire in a single stimulation session is limited by these practical considerations, an alternative option to increase the number of MEPs would be to increase the number of sessions per participant and then pool the MEPs across sessions.

In future experiments, tACS will instead be applied at a frequency that is naturally present in the motor cortex during wakefulness, such as the sensorimotor mu (μ) rhythm (8–13 Hz; Antal et al., 2008; Wach et al., 2013; Gundlach et al., 2017; Thies et al., 2018; Feurra et al., 2019; Madsen et al., 2019). This will also allow us to determine if the excitatory effect observed in the present experiment is specific to SO tACS or if similar effects are observed for other stimulation frequencies.

Alternatively, because the present experiment did not include a negative control stimulation condition for SO tACS (e.g., sham stimulation), it is theoretically possible that the observed increase in motor cortical excitability is simply a time-dependent effect and not mediated by tACS and this is a limitation of the experiment. However, this seems highly unlikely given that a recent meta-analysis by Dissanayaka et al. (2018) reported no significant effects of sham tES on cortical excitability compared to baseline, even up to 90 min following sham tES (Moliadze et al., 2010, 2012; Chaieb et al., 2011). Although only some of the assessed tES studies specifically investigated tACS, all of the studies used a comparable fade-in, short stimulation, fade-out (FISSFO) sham condition, and thus, they can all be used to make inferences about the likely tACS-free changes in MEP amplitude (i.e., solely due to time). This provides a compelling null comparator for the significant increase in MEP amplitude by SO tACS.

Although the underlying cause of the observed increase in MEP amplitude cannot be concluded from the present results, the lack of phase-specific entrainment suggests that this excitatory effect may instead be driven by plasticity-related mechanisms, such as spike-timing dependent plasticity (STDP; Veniero et al., 2015; Vossen et al., 2015). In the STDP model, even a slight mismatch between the stimulation frequency and an individual’s spontaneous peak frequency could influence the direction of any induced changes, which may explain the heterogeneity of tACS aftereffects across studies (Veniero et al., 2015). Tests of this model should therefore tailor stimulation frequency to each participant’s individual peak frequency rather than use a standard frequency such as was used in the present study.

It is important to note that entrainment and plastic-like effects induced by tACS are not mutually exclusive (Vosskuhl et al., 2018). In fact, Helfrich et al. (2014a, b) found the magnitude of induced aftereffects to be positively correlated with the magnitude of online entrainment and also demonstrated that online effects occurred within a narrow frequency range whilst offline effects occurred across a broader band around the frequency of tACS. This suggests that whilst online effects may be explained by entrainment, sustained aftereffects may be better explained by entrainment-mediated changes to network strength, which then oscillates close (but not necessarily equal) to the frequency of stimulation.

Elucidation of the mechanisms underlying the online and offline effects of tACS will better its therapeutic applications. For example, the ability to induce lasting plastic changes in the motor cortex using tACS may improve the effectiveness of existing rehabilitation for neurological conditions where motor function is compromised.

## Conclusion

In summary, the significant increase in TMS-induced MEP amplitudes from pre- to post-SO tACS as well as from the 1st to the 3rd SO tACS block suggests that, similar to previously reported excitability increases induced by anodal SO tDCS, SO tACS had a facilitatory effect on motor cortical excitability that outlasted the stimulation period. Importantly, the present findings suggest that these motor cortical excitability increases are not simply due to anodal stimulation. However, given the acute effects of SO tACS were independent of phase, this study does not support entrainment of endogenous slow oscillations as an underlying mechanism for this excitatory effect.

## Data Availability Statement

The datasets presented in this study can be found in online repositories. The names of the repository/repositories and accession number(s) can be found below: https://doi.org/10.17605/OSF.IO/DAV4T, Open Science Framework, Reference “SO tACS 2019.”

## Ethics Statement

The studies involving human participants were reviewed and approved by The University of Queensland Human Research Ethics Committee. The patients/participants provided their written informed consent to participate in this study.

## Author Contributions

AG: conceptualization, methodology, software, formal analysis, investigation, data curation, writing–original draft, and visualization. NB: conceptualization, methodology, software, data curation, writing–review and editing, visualization, and supervision. MS: conceptualization, methodology, validation, resources, writing–review and editing, project administration, funding acquisition, and supervision. All authors contributed to the article and approved the submitted version.

## Conflict of Interest

The authors declare that the research was conducted in the absence of any commercial or financial relationships that could be construed as a potential conflict of interest.

## Publisher’s Note

All claims expressed in this article are solely those of the authors and do not necessarily represent those of their affiliated organizations, or those of the publisher, the editors and the reviewers. Any product that may be evaluated in this article, or claim that may be made by its manufacturer, is not guaranteed or endorsed by the publisher.

## Supplementary Material

Supplementary Figure S1 Fitted sinusoidal models for each participant’s late online MEPs. Each scatter plot represents one participant (*n* = 30), with blue dots (*n* = 60) representing individual MEP amplitudes (*y*-axis) sorted according to tACS phase (*x*-axis). Orange dots represent the sinusoidal model fitted to each scatter plot. A permutation analysis across all participants revealed no significant modulation of late online MEP amplitudes with respect to tACS phase (*p* = 0.81). Note there was a “gap” in phase values for the first 3 participants due to an error in the MATLAB script that determined the timing of TMS delivery, but this error was addressed after the 3rd participant and the phase gap is not present in any of the other participants.

Supplementary Figure S2 Histograms of fitted sinusoidal model amplitudes for each position. Bars represent the number of participants with fitted sinusoidal model amplitudes within the range specified on the *x*-axis for **(A)** early online, **(B)** late online, **(C)** early offline, and **(D)** late offline MEPs.

## Abbreviations

tES: transcranial electrical stimulation
tACS: transcranial alternating current stimulation
tDCS: transcranial direct current stimulation
SO: slow oscillatory
TMS: transcranial magnetic stimulation
EEG: electroencephalography
EMG: electromyography
MEP: motor evoked potential
M1_HAND_: hand area of the primary motor cortex
APB: *abductor pollicis brevis;*
FISSFO: fade-inshort, stimulation, fade-out
STDP: spike-timing dependent plasticity.
