# Supplementary material for: Effects of Slow Oscillatory Transcranial Alternating Current Stimulation on Motor Cortical Excitability Assessed by Transcranial Magnetic Stimulation
Source: Front Hum Neurosci. 2021 Sep 13;15:726604. doi: 10.3389/fnhum.2021.726604 (PMC8473706; doi:10.3389/fnhum.2021.726604)
Supplement: Supplementary Figure S1 — Fitted sinusoidal models for each participant’s late online MEPs. Each scatter plot represents one participant (n = 30), with blue dots (n = 60) representing individual MEP amplitudes (y-axis) sorted according to tACS phase (x-axis). Orange dots represent the sinusoidal model fitted to each scatter plot. A permutation analysis across all participants revealed no significant modulation of late online MEP amplitudes with respect to tACS phase (p = 0.81). Note there was a “gap” in phase values for the first 3 participants due to an error in the MATLAB script that determined the timing of TMS delivery, but this error was addressed after the 3rd participant and the phase gap is not present in any of the other participants. [file Data_Sheet_1.docx]

#
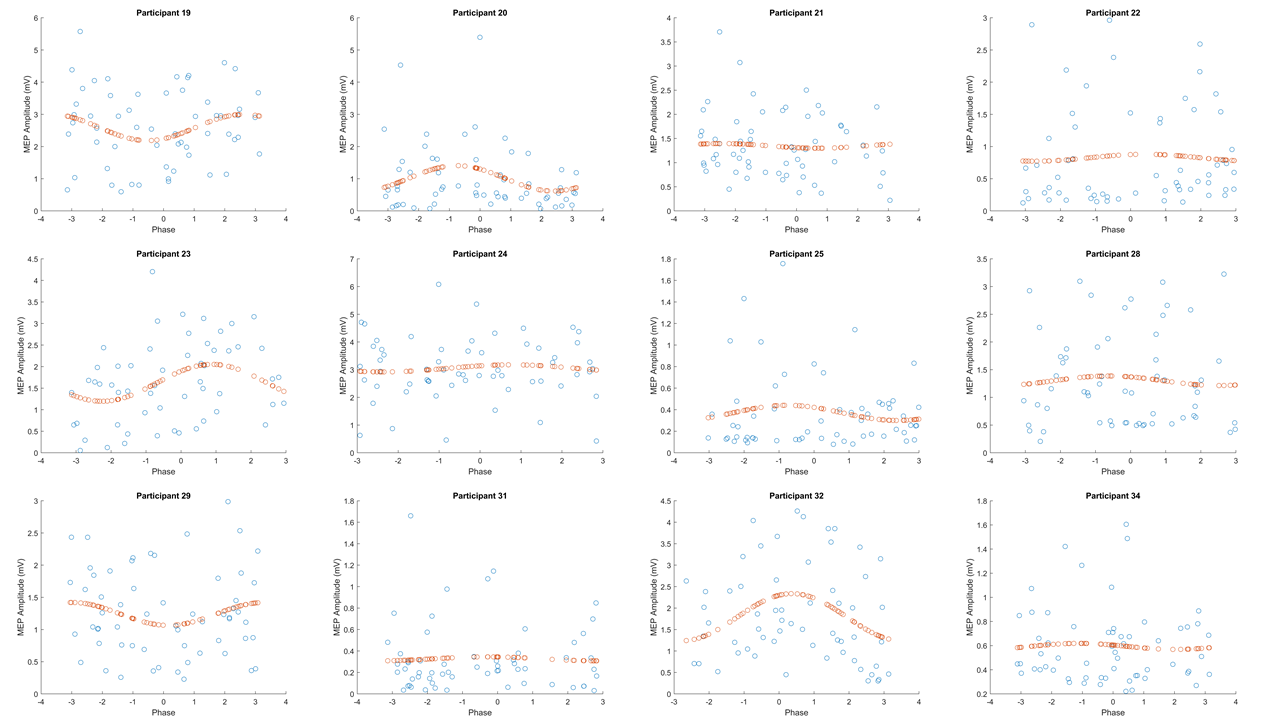

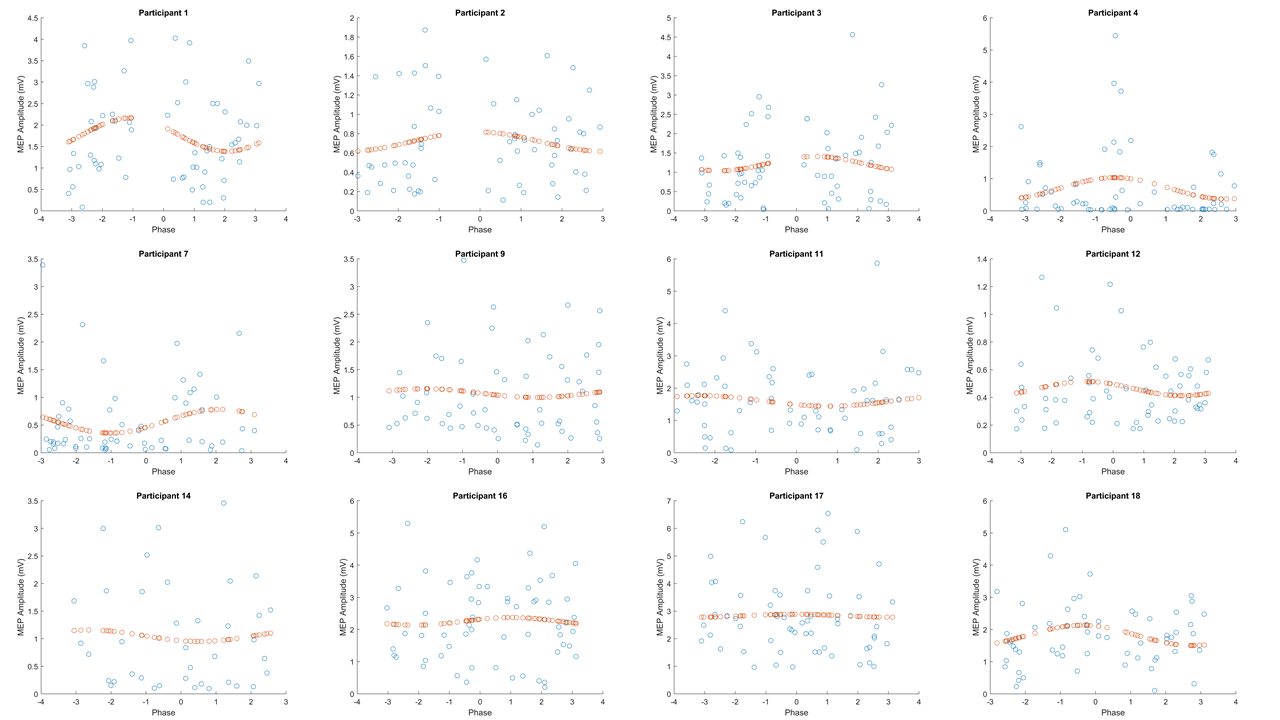


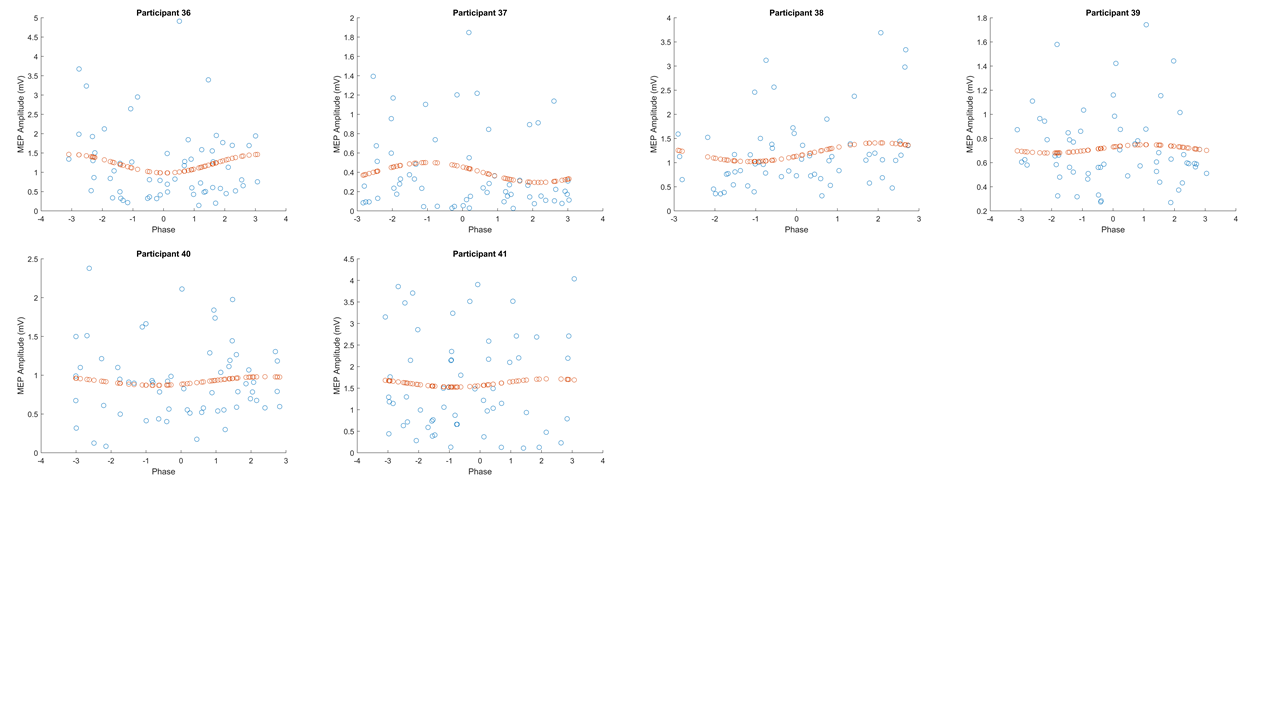


**Supplementary Figure 1.** **Fitted Sinusoidal Models for Each Participant's Late Online MEPs.** Each scatter plot represents one participant (n = 30), with blue dots (n = 60) representing individual MEP amplitudes (y-axis) sorted according to tACS phase (x-axis). Orange dots represent the sinusoidal model fitted to each scatter plot. A permutation analysis across all participants revealed no significant modulation of late online MEP amplitudes with respect to tACS phase (p = 0.81). Note there was a “gap” in phase values for the first 3 participants due to an error in the MATLAB script that determined the timing of TMS delivery, but this error was addressed after the 3rd participant and the phase gap is not present in any of the other participants.
