# Supplementary material for: Effects of Slow Oscillatory Transcranial Alternating Current Stimulation on Motor Cortical Excitability Assessed by Transcranial Magnetic Stimulation
Source: Front Hum Neurosci. 2021 Sep 13;15:726604. doi: 10.3389/fnhum.2021.726604 (PMC8473706; doi:10.3389/fnhum.2021.726604)
Supplement: Supplementary Figure S2 — Histograms of fitted sinusoidal model amplitudes for each position. Bars represent the number of participants with fitted sinusoidal model amplitudes within the range specified on the x-axis for (A) early online, (B) late online, (C) early offline, and (D) late offline MEPs. [file Data_Sheet_2.docx]

*
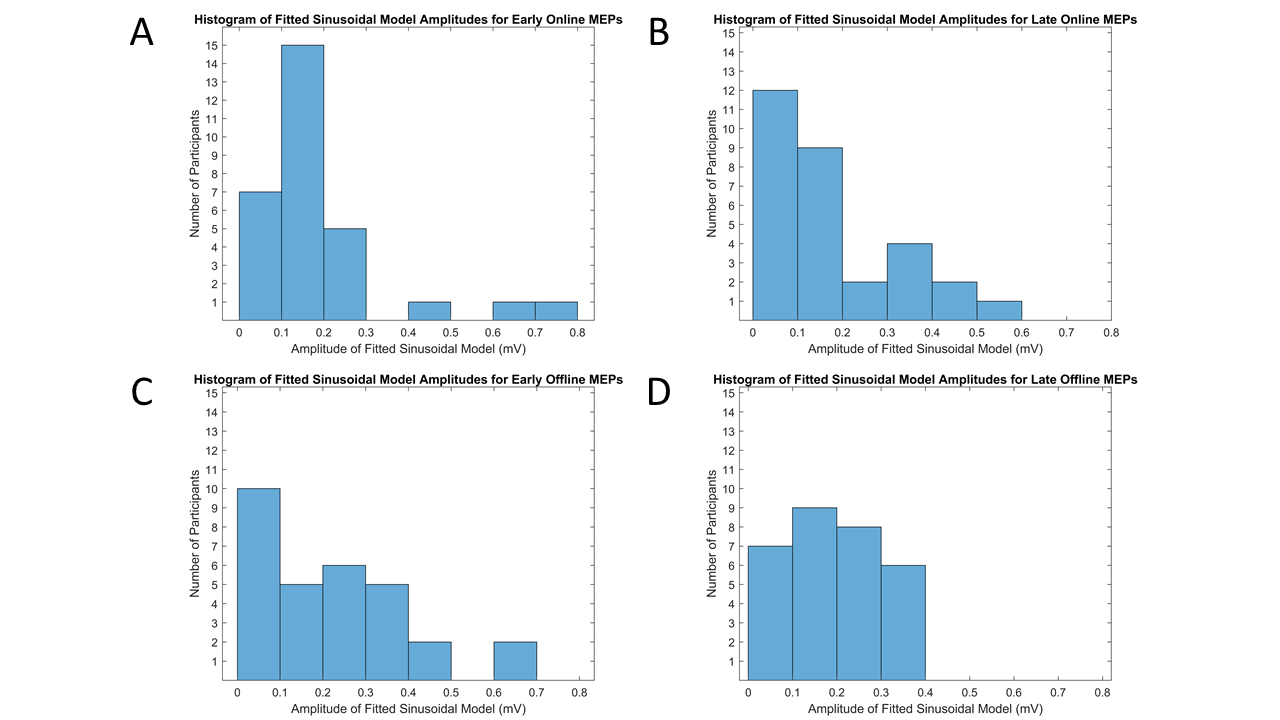
*

**Supplementary Figure 2. Histograms of Fitted Sinusoidal Model Amplitudes for Each Position.** Bars represent the number of participants with fitted sinusoidal model amplitudes within the range specified on the x-axis for (A) early online, (B) late online, (C) early offline, and (D) late offline MEPs.
